# Valence-Dependent Coupling of Prefrontal-Amygdala Effective Connectivity during Facial Affect Processing

**DOI:** 10.1523/ENEURO.0079-19.2019

**Published:** 2019-07-25

**Authors:** David Willinger, Iliana I. Karipidis, Selina Beltrani, Sarah V. Di Pietro, Ronald Sladky, Susanne Walitza, Philipp Stämpfli, Silvia Brem

**Affiliations:** 1 Department of Child and Adolescent Psychiatry and Psychotherapy, University Hospital of Psychiatry Zurich, University of Zurich, Zurich 8032, Switzerland; 2 Department of Psychiatry, Psychotherapy and Psychosomatics, University Hospital of Psychiatry Zurich, University of Zurich, Zurich 8008, Switzerland; 3 Neuroscience Center Zurich, University of Zurich and ETH Zurich, Zurich 8057, Switzerland; 4 Center for Interdisciplinary Brain Sciences Research, Stanford University School of Medicine, Stanford, CA 94305; 5 Social, Cognitive and Affective Neuroscience Unit, Department of Basic Psychological Research and Research Methods, Faculty of Psychology, University of Vienna, Vienna 1010, Austria

**Keywords:** amygdala, DCM, emotional valence, fMRI, prefrontal cortex

## Abstract

Despite the importance of the prefrontal-amygdala (AMY) network for emotion processing, valence-dependent coupling within this network remains elusive. In this study, we assessed the effect of emotional valence on brain activity and effective connectivity. We tested which functional pathways within the prefrontal-AMY network are specifically engaged during the processing of emotional valence. Thirty-three healthy adults were examined with functional magnetic resonance imaging while performing a dynamic faces and dynamic shapes matching task. The valence of the facial expressions varied systematically between positive, negative, and neutral across the task. Functional contrasts determined core areas of the emotion processing circuitry, comprising the medial prefrontal cortex (MPFC), the right lateral prefrontal cortex (LPFC), the AMY, and the right fusiform face area (FFA). Dynamic causal modelling demonstrated that the bidirectional coupling within the prefrontal-AMY circuitry is modulated by emotional valence. Additionally, Bayesian model averaging showed significant bottom-up connectivity from the AMY to the MPFC during negative and neutral, but not positive, valence. Thus, our study provides strong evidence for alterations of bottom-up coupling within the prefrontal-AMY network as a function of emotional valence. Thereby our results not only advance the understanding of the human prefrontal-AMY circuitry in varying valence context, but, moreover, provide a model to examine mechanisms of valence-sensitive emotional dysregulation in neuropsychiatric disorders.

## Significance Statement

Recent neuroimaging studies have emphasized the importance of valence-sensitivity within the prefrontal-amygdala (AMY) network during emotion processing. Yet, it remains elusive which specific pathways are involved in processing affective information, and how this information is integrated in the brain’s network. In particular, the AMY’s role in signaling valence information to the cortex is subject to ongoing discussions. Moreover, as aberrant brain function has been found in the AMY and the prefrontal cortex in various debilitating psychiatric disorders, understanding the mechanisms of processing emotional stimuli with different valence (positive, negative, neutral) is particularly relevant for the field. Our findings indicate changes in coupling strength as a function of emotional valence within the prefrontal-AMY network.

## Introduction

The prefrontal-amygdala (AMY) network plays a pivotal role in adapting human behavior to constantly changing environmental demands. Previous neuroimaging research has emphasized the importance of interactions between the prefrontal cortex and the AMY during affective processing (Phillips et al., 2008; Ochsner et al., 2009) and has tried to disentangle bottom-up from top-down mechanisms of emotion processes (Ochsner et al., 2009; Whalen et al., 2013; Comte et al., 2016; Pessoa, 2017). Emotional salience related to the perceptual properties of a stimulus, as mediated by emotional faces, is thought to be propagated from the AMY to the prefrontal cortex via bottom-up connections (McRae et al., 2012). It has long been recognized that the AMY plays a crucial role in immediate, automatic processing of emotional information and the modulation of attention (Anderson et al., 2003; Phelps, 2006; Ochsner et al., 2009). Conversely, top-down signaling during emotion processing has been attributed to different forms of emotion regulation, where the lateral prefrontal cortex (LPFC) supports top-down evaluation of contextual significance and altering of the affective response by exerting cognitive control over limbic regions (Ochsner and Gross, 2005; Quirk and Beer, 2006; Dima et al., 2011), even without explicit instruction (Drabant et al., 2009).

This coupling between the LPFC and the AMY is central to theoretical models of emotion processing. Nevertheless, emotion processing involves complex interactions between AMY driven bottom-up salience processing, and top-down contextualization and evaluation of stimuli, supported by the LPFC, whose strength and directions can differ substantially depending on context, e.g., emotional valence or task demands (Kim et al., 2004; Pessoa, 2017). Lately, this has led to new conceptions, where emotion processing is strongly interwoven with other mental entities that constitute cognition (e.g., memory or attention), and relies on dynamic, context-sensitive interactions of top-down and bottom-up processes (Pessoa, 2017).

Given that structural connections between the LPFC and the AMY are sparse (Ray and Zald, 2012), regulatory signals from the LPFC are likely mediated to the AMY via the medial prefrontal cortex (MPFC). The MPFC is situated perfectly to pass on top-down appraisal and regulation signals to limbic structures as it shares rich bidirectional connections with the LPFC and the AMY (Price, 2005; Ray and Zald, 2012). As such, the MPFC has not only been implicated in regulation of emotional responses, in particular to aversive stimuli, but also in integrating affective and contextual information, i.e., bottom-up and top-down signals, to support generation of affective meaning (Delgado et al., 2008; Ochsner et al., 2009; Roy et al., 2012; Etkin et al., 2015; Silvers et al., 2015; Comte et al., 2016; Lindquist et al., 2016). However, the valence-dependent coupling between regions comprising the emotion processing circuitry is only poorly understood. Particularly, the role of the AMY in encoding valence is still debated (Ball et al., 2009; Jin et al., 2015), and thus far, valence-dependent alterations of directed coupling between the AMY and the prefrontal cortex during emotion processing has not been investigated, despite it being strongly implicated in psychopathology (Dichter et al., 2009; Disner et al., 2011; Sladky et al., 2015b).

In this study, we used a novel dynamic face-matching and shape-matching task to investigate the effect of valence of facial expressions on effective connectivity within the prefrontal-AMY circuitry in 33 healthy adults. Dynamic faces have a higher ecological validity than traditionally used static faces and have been shown to elicit strong responses in brain networks of interest in several fMRI paradigms (Arsalidou et al., 2011; Kessler et al., 2011; Furl et al., 2015). Negatively, neutrally, and positively valenced facial expressions were used to examine the effect of valence on the prefrontal-AMY network, shapes served as a control condition.

In agreement with previous findings using static face processing tasks (Gläscher et al., 2004; Vuilleumier et al., 2004) or affective pictures (Urry et al., 2006), we expected an increased activation for negative valence in bilateral AMYs and the MPFC compared to the neutral and positive valence conditions of our dynamic paradigm. Moreover, dynamic causal modeling (DCM) was used to clarify the contextual influence of valence on the functional architecture of the emotion-processing network. Thus, we investigated whether valence of facial affect modulates effective connectivity within the hierarchical network architecture in a bottom-up, a top-down, or, as recently suggested (Pessoa, 2017), a bidirectional manner. Given the role of the MPFC in integrating context and salience to shape emotional responses (Roy et al., 2012; Etkin et al., 2015), we hypothesized that affective information would modulate bidirectional connections between MPFC and AMY, as well as between MPFC and LPFC.

## Materials and Methods

### Subjects

A group of 33 healthy volunteers (mean age and SD, 27.4 ± 5.2 years, 24 females and nine males, 30 right handed and three left handed) was recruited for this study. Inclusion criteria were age of 18–45 years and signed informed consent. Exclusion criteria included any MRI contraindication, pregnancy, a history of brain injury, psychiatric disorders, other major medical illnesses, and drug abuse. No subject reported any past or current psychiatric disorder. During scanning, none of the subjects exceeded our motion threshold of a mean framewise displacement (Power et al., 2012) of 0.5 mm (mean 0.14 ± 0.09 mm).

This study was approved by the ethics committee of the Kanton Zurich and was conducted in accordance with the Declaration of Helsinki.

### Experimental design

**Figure 1.**
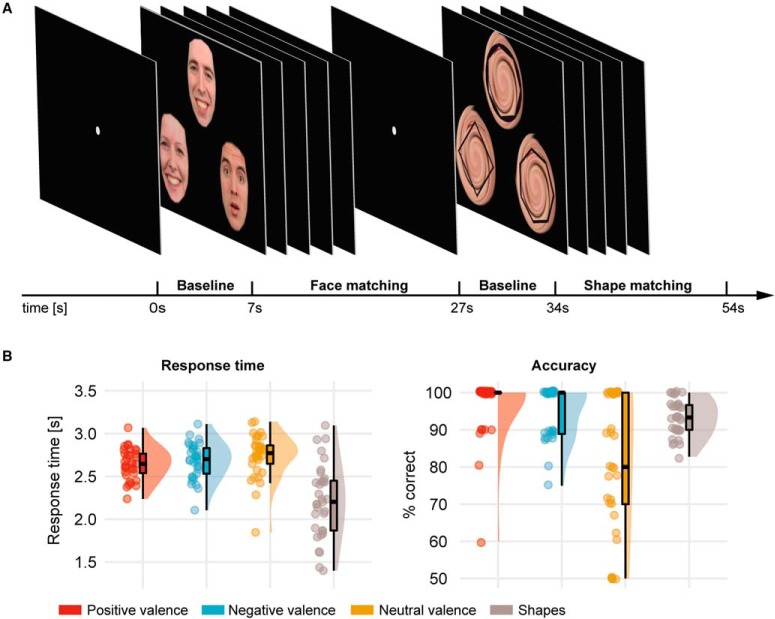
***A***, Experimental design of the study. All participants were presented with triplets of emotional faces (blocks of positive, negative, and neutral valence) and shapes (polygons). The task comprised matching the dynamic target image on top with one of the two static probe images at the bottom by the emotion (faces, with distinct emotional expressions for the static probe images) or number of vertices (shapes). ***B***, Behavioral results of the dynamic face-matching and shape-matching paradigm. RTs were comparable across different valence conditions. Response accuracy in trials with neutral faces was lower than in trials with positive or negative faces.

All participants completed a 6-min fMRI dynamic face-matching and shape-matching task (Fig. 1*A*), which is based on the static task used by Hariri et al. (2002).

Face-matching and shape-matching blocks had a length of 20 s and were presented alternatingly. Each of the 12 blocks (six face and six shape blocks) consisted of five trials with a length of 4 s. In each trial, participants were presented with a dynamic target item and two static probe images below it, one of which matched with the target item with regard to shape or facial expression. Subjects were instructed to match either the left or the right static item at the bottom with the dynamic item on top and to press either the left or the right button with their dominant hand on a response-pad to indicate their choice as soon as they recognized which probe matched.

We used face images from the Radboud face database (Langner et al., 2010), including the faces of 38 white actors (19 females). In total, we presented six face-matching blocks (30 trials, 10 trials per valence), two positively valenced, including happy, surprised and neutral faces, two negatively valenced, sad and disgusted faces, and two neutrally valenced, neutral and surprised faces (for a view on surprise as neutrally valenced, see Sander and Scherer, 2014; Soriano Salinas et al., 2015). In the negatively valenced condition, we used sad and disgusted faces instead of widely used fearful faces to capture negative emotion processing not related to arousal (Remmington et al., 2000; Trautmann et al., 2009; Langner et al., 2010). To construct the stimuli for the positive condition, we used the inherently positive valence “happy,” and included faces with neutrally valenced expressions, surprised and neutral, for the face-matching task. This formed overall positively valenced stimulus triplets in all trials, as ambiguous faces (i.e., surprised or neutral) have been shown to be perceived more positive when being presented within a positive context (Neta et al., 2011). Importantly, in each trial of the positive condition, subjects were presented with at least one happy face, either as target or probe. Before the study, we established that a positive condition comprising neutral and positive faces only had lower task difficulty than the other two conditions (neutral and negative blocks). The selection of both, neutral and surprised faces, in the positive condition rendered the task difficulty across conditions comparable. Based on the face scores determined by Langner et al. (2010), the average valence (mean ± SD) of the faces used was 3.55 ± 0.08 for the positive condition, 2.94 ± 0.04 for the neutral condition, and 2.01 ± 0.04 for the negative condition, where 1 represents the most negative and 5 the most positive possible valence rating. Shapes were superimposed to a whirled face in six shape-matching blocks.

We adapted the original task used by Hariri et al. (2002) using a dynamic video sequence of the target emotion or shape to make our task ecologically more valid. Target faces on top were morphed from 0% (neutral faces) to 100% (emotional faces) within the trial time of 4 s. Neutral dynamic target stimuli were morphed to surprised emotion from 0% to 30% intensity and back to 0% intensity to introduce neutrally valenced facial motion. Similarly, during the shape-matching task, the target shape was morphed from a round circle into a polygon with three to eight vertices. During face-matching and shape-matching, probe images on the bottom always remained static. After the button press, the video sequence continued until the end of the morphing sequence of the target face or shape (100% morphed, 4 s). We ensured correct understanding of the task by familiarizing the subjects with the task outside the scanner.

### Data acquisition and preprocessing

All MRI recordings were performed on a Philips Achieva 3 Tesla scanner (Philips Medical Systems) using a 32-channel head coil. Functional images were acquired with a multiband echoplanar images (EPIs) sequence (175 volumes, repetition time TR = 2 s, echo time TE = 35 ms, 15° tilted downwards of AC-PC, 54 slices, voxel size = 2.0 × 2.0 × 2.5 mm^3^, matrix size = 96 × 94 px, flip angle = 80°, no gap, SENSE-factor = 2, MB-factor = 2). Before the actual data acquisition, we acquired five dummy scans to establish steady-state conditions. After performing the task, we acquired a T1-weighted anatomic image for each subject that was used for coregistration and normalization of functional data with a 3D magnetization-prepared rapid gradient-echo sequence (MP-RAGE; time between two inversion pulses = 2484 ms, inversion time TI = 900 ms, inter-echo delay = 6.7 ms, aligned at AC-PC, flip angle = 9°, voxel size = 1.05 × 1.05 × 1.2 mm^3^, field of view = 270 × 354 mm^2^, 170 sagittal slices).

Preprocessing of the images included slice-timing correction, realignment, coregistration, and segmentation. Normalization to the Montreal Neurologic Institute (MNI)-152 template space was performed using the deformations derived from the segmentation step. In addition, preprocessing included resampling to 2-mm isometric voxels, and smoothing with a 6-mm full-width-half-maximum (FWHM) Gaussian kernel. All steps were performed using SPM12 (7219) software.

### Behavioral analysis

To analyze the behavioral data, we conducted a repeated measures ANOVA to test for effects between valence conditions and paired *t* tests to test for any difference in performance between face and shape matching (Table 1). Trials without response or a response time (RT) <100 ms were excluded from the behavioral analysis (3.9% of all trials).

### Whole-brain analysis

The first-level analysis was conducted by building a general linear model using the individual onset and length of each trial (4 s) for face-matching and shape-matching convolved with the canonical hemodynamic response function as implemented in SPM12. To model the valence of faces, we added three regressors for each of the respective conditions. The final GLM for the whole-brain analysis included five regressors of interest: a regressor for all faces, three parametric modulation regressors for each valence, that is for positive, negative, and neutral faces, and one regressor for shapes. The regressor “all faces” included 30 events, while parametric modulation regressors modeling positive, negative, and neutral conditions comprised 10 events each. The regressor “shapes” included 30 events. In addition, we added the six realignment parameters derived from preprocessing as nuisance regressors.

The main effect of our task (face-matching > shape-matching) was investigated with a one-sample *t* test using the respective contrast files of each subject. To examine the effect of valence, we performed an *F* test in a second-level repeated measures ANOVA design across the positive, negative, and neutral valence conditions. For both analyses the cluster-based family-wise error corrected significance threshold was set to *p*_FWEc_ = 0.05, the uncorrected voxel-wise cluster-defining threshold was set to *p*_CTD_ = 0.001. Labels of resulting brain regions were determined using the SPM Anatomy Toolbox (Eickhoff et al., 2007).

### Dynamic causal modeling (DCM)

DCM is a hypothesis-driven Bayesian model comparison procedure for inferring effective connectivity between brain regions (Friston et al., 2003). DCM allows for the creation of different models to investigate the directed interactions of specific brain regions under experimentally controlled perturbations. These interactions are modeled at the neuronal level and related to the observable measurement via a hemodynamic forward model (Buxton et al., 1998). Importantly, it allows for estimation of endogenous coupling and context-specific, modulatory coupling (Friston et al., 2003; Penny et al., 2004). The neural model is given by the neural state equation

$$\frac{dz}{dt}=\left(A+\sum u_{j}B^{j}\right)z+Cu$$

in which the vector *z* represents the time series of the neural signal in a given region of interest and *u* represents the experimental inputs (1 … *j*). Intrinsic (endogenous) coupling parameters between regions are stored in matrix *A*, modulatory parameters for a stimulus *u_j_* are stored in matrix *B*, and direct driving inputs for regions are described in matrix *C*.

#### Regional time series extraction

In our study, we focused on the analysis of an emotion processing network model comprising four regions, whose adequacy has been demonstrated in previous studies (Almeida et al., 2009a, 2011; Sladky et al., 2015a). In particular, we included (1) the ventrolateral part of the LPFC that is associated with emotion regulation (Hariri et al., 2003; Morris et al., 2012; Wagner and Heatherton, 2013); (2) the MPFC that is involved in integrating affective and contextual information, valence processing (Roy et al., 2012), and autonomous emotion regulation (Phillips et al., 2008); (3) the AMY for its role in salience detection and facial emotion processing (Phelps, 2006); and (4) the fusiform face area (FFA) as part of the visual system, that is sensitive to faces (Kanwisher et al., 1997).

The selection and functional localization of our volumes of interest (VOI) in the AMY-prefrontal network was guided by the results of the second-level group analyses (Table 2), similar to previous work (Hauser et al., 2014; Sladky et al., 2015a). For the AMY, the LPFC, and the FFA, we specified a spherical search volume at the peak of the face-matching > shape-matching contrast: AMY *x* = 21, *y* = –10, *z* = –14 mm (MNI); the right LPFC *x* = 47, *y* = 30, *z* = 8 mm (MNI); the right FFA *x* = 41, *y* = –44, *z* = –22 mm (MNI). In addition, we defined a search volume for the MPFC at the peak of the main effect of valence in the second-level ANOVA comparing positive, negative, and neutral valence conditions [*x* = 3, *y* = 50, *z* = –2 mm (MNI)]. The individual VOI center coordinates were restricted to not differ >12 mm (corresponding to twice the FWHM of the smoothing kernel) from the group maximum to ensure comparability between subjects.

Subjects’ individual spherical VOIs were centered at the individual peaks (*r* = 6 mm, *p* < 0.05, uncorrected) in the respective contrast and the first eigenvariate was extracted as summary statistic for all active voxels within the VOI. One subject was excluded from the DCM analysis, because we did not find any active voxels in the LPFC for the chosen threshold. We restricted our analysis to the right hemisphere, as previous studies suggested that it preferentially engages in processing of nonverbal emotional cues, such as emotional faces (Puce et al., 1996; Kanwisher et al., 1997; Anderson et al., 2003; Ochsner et al., 2004; Fairhall and Ishai, 2007; Sladky et al., 2015a).

#### Model space

We assumed bidirectional connection between MPFC and AMY, and MPFC and LPFC. Although there is evidence that direct connections between LPFC and AMY are only very sparse (Ray and Zald, 2012), it is possible that they exert influence via indirect pathways over each other. Hence, we included models with all possible intrinsic connectivity patterns between the LPFC and the AMY in the model space. In addition, we specified bidirectional intrinsic connections between the FFA and the LPFC and the FFA and the AMY, respectively. Modulation by valence was varied systematically across connections between MPFC and LPFC, and MPFC and AMY in all possible modulation patterns, spanning a model space of 256 models.

For the DCM analysis we specified a second GLM that included five regressors of interest (all stimuli, all faces, positive faces, negative faces, and shapes) and the six realignment parameters as nuisance regressors. The “all stimuli” regressor included 60 trials, “all faces” included all 30 face events, regressors modeling positive, negative comprised 10 events each, and the “shape” regressor included all 30 shape-matching trials. The “all faces” regressor served as driving input of the FFA.

We performed random-effects family-wise Bayesian model selection (BMS; Penny et al., 2010) as implemented in SPM12 to compute the expected posterior probabilities and the exceedance probabilities of model families within our sample. For model comparison, the BMS procedure uses the free energy that is a lower-bound approximation to the log-model evidence that accounts for both model accuracy and model complexity (Penny et al., 2004; Penny, 2012).

**Figure 2.**
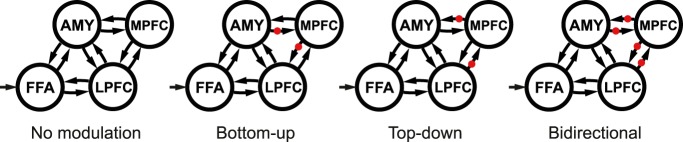
DCM model specification. We specified 256 models and grouped them into four families, depending on the location of the modulations of positive and negative valence. The modulations are depicted as red dots on the connections. In each family, all possible combinations of modulations were grouped together yielding one model with no modulation, 15 models with bottom-up modulations, 15 models with top-down modulations, and 225 models with bidirectional modulations. All faces were the driving input to the FFA.

To test different functional architectures of contextual modulation, we created four different families of models (Fig. 2). These model families differed in terms of connections on which emotional valence modulated effective connectivity. We created families with no contextual (valence) modulations (one model), bottom-up modulations (15 models), top-down modulations (15 models), and bidirectional modulations (225 models).

We used Bayesian model averaging (BMA) across models to make further inferences on the significance of connections and modulation by valence (Penny et al., 2010). BMA allows for averaging the parameters while they are weighted by the posterior probability of the model and thereby accounting for the uncertainty of individual models (Stephan et al., 2010).

Subsequent one-sample *t* tests of averaged parameter estimates were conducted in MATLAB (MATLAB and Statistics Toolbox Release 2017a, The MathWorks, Inc.). We accounted for multiple *t* tests of the connectivity parameters by using the procedure of Benjamini and Hochberg (1995) to control the false discovery rate (FDR; adjusted *p*_FDRc_ < 0.05).

## Results

### Behavioral analysis

The behavioral analysis of the RT and the accuracy across different conditions is summarized in Table 1 and depicted in Figure 1*B*.

**Table 1.** Behavioral results of the analysis of the behavioral data

	Positive valence	Negative valence	Neutral valence	Shapes
Accuracy	96.6 ± 8.2%	94.9 ± 7.0%	81.0 ± 18.4%	93.5 ± 6.1%
RT	2.65 ± 0.18 s	2.68 ± 0.21 s	2.74 ± 0.26 s	2.22 ± 0.12 s

Mean ± SD across all subjects (*n* = 33).

Responses during shape-matching were significantly faster than during face-matching (*t*_(32)_ = 5.97, *p* < 10^–5^). Accuracy (% correct) did not differ significantly between face-matching and shape-matching (*t*_(32)_ = –2.01, *p* = 0.053). There was a main effect of valence on accuracy for the three valence conditions (*F*_(2.32)_ = 24.02, *p* < 10^–7^). Pairwise comparisons indicated that accuracy during trials with neutral faces was lower than trials with positive (*t*_(32)_ = 5.56, *p* < 10^–5^) and negative faces (*t*_(32)_ = 4.74, *p* < 0.0001), suggesting a higher difficulty in matching faces of the neutral condition. Positive and negative face conditions did not differ in accuracy (*t*_(32)_ = 1.28, *p* = 0.21). A similar accuracy pattern was reported in previous work (Aybek et al., 2015). One-sample *t* tests across positive (*t*_(32)_ = 32.69, *p* < 10^–25^), negative (*t*_(32)_ = 36.92, *p* < 10^–275^) and neutral valence (*t*_(32)_ = 9.69, *p* < 10^–10^) showed that accuracies were well beyond chance level (50%). Importantly, in a repeated measures ANOVA we did not find any significant differences in RTs across valence conditions (*F*_(2.32)_ = 1.79, *p* = 0.175).

### Whole-brain results

**Figure 3.**
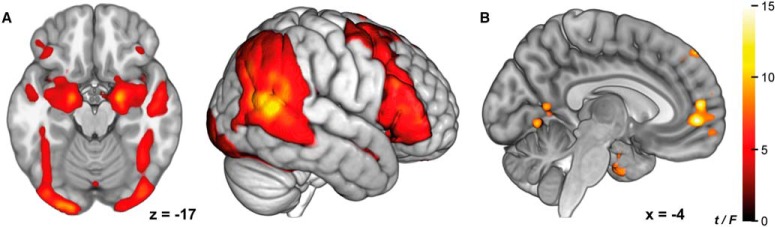
Whole-brain group analyses. ***A***, Main effect of task face-matching > shape-matching. ***B***, Main effect of valence in the MPFC. Both images thresholded at *p*_FWEc_ < 0.05, with a voxel-wise cluster-defining threshold of *p*_CDT_ < 0.001, *n* = 33. Color is mapped to *t* values (***A***) and *F* values (***B***).

The dynamic face-matching and shape-matching task showed a significant effect of task (face-matching > shape-matching) in brain regions commonly recruited during face processing (Fusar-Poli et al., 2009), including the AMY, the fusiform gyrus, the LPFC, the middle and superior temporal gyrus (Fig. 3*A*; Table 2). A repeated measures ANOVA (Fig. 3*B*) across valence conditions using the respective contrast images revealed a main effect of valence in the MPFC (*F*_(2.32)_ = 17.54, *p*_FWEc_ < 10^–7^), the right medial temporal lobe (*F*_(2.32)_ = 18.14, *p*_FWEc_ = 0.043), the superior temporal gyrus (*F*_(2.32)_ = 16.13, *p*_FWEc_ = 0.002), the left medial temporal lobe (*F*_(2.32)_ = 15.34, *p*_FWEc_ < 10^–5^), the left cerebellum (*F*_(2.32)_ = 14.79, *p*_FWEc_ = 0.041), the left AMY (*F*_(2.32)_ = 14.72, *p*_FWEc_ = 0.009), and the right parahippocampal gyrus (*F*_(2.32)_ = 14.70, *p*_FWEc_ = 0.001). Post-hoc *t* tests showed that the effect in the MPFC was driven by negative valence (Table 2).

**Table 2.** Results of the group analysis (*n* = 33)

	MNI coordinates (mm)			Cluster level		Peak
Brain region	*x*	*y*	*z*	*p*_FWEc_	k	Z
Face-matching > shape-matching						
R middle temporal gyrus	55	–42	6	*p* < 0.0001	8605	7.54
R AMY	19	–8	–16			6.87
R inferior occipital gyrus	25	–94	–4	*p* < 0.0001	2339	7.37
R fusiform gyrus	41	–44	–22			6.54
L lingual gyrus	–21	–96	–14	*p* < 0.0001	2143	7.30
L fusiform gyrus	–41	–50	–22			6.61
R inferior frontal gyrus	47	30	8	*p* < 0.0001	5966	7.24
L middle temporal gyrus	–53	–60	10	*p* < 0.0001	5096	6.91
L inferior frontal gyrus	–45	34	2	*p* < 0.0001	9479	6.63
L AMY	–19	–8	–14	*p* < 0.0001	1110	6.52
R precuneus	9	–58	40	*p* < 0.0001	861	6.50
R inferior temporal gyrus	43	–12	–42	*p* < 0.0001	569	6.18
L inferior temporal gyrus	–43	–16	–44	*p* < 0.0001	478	6.12
L cerebellum	–17	–74	–34	*p* < 0.0001	604	5.78
R middle frontal gyrus	27	50	6	*p* = 0.0018	229	4.20
Effect of valence (ANOVA)						
R medial temporal pole	45	10	–36	*p* = 0.043	103	5.06
L anterior cingulate cortex	–3	50	–2	*p* < 0.0001	615	4.98
R superior temporal gyrus	49	–6	–4	*p* = 0.002	185	4.77
L medial temporal pole	–43	14	–34	*p* < 0.0001	350	4.65
L lingual gyrus	–17	–66	–4	*p* = 0.041	104	4.56
L AMY	–19	–6	–24	*p* = 0.009	142	4.55
R parahippocampal gyrus	23	–16	–20	*p* = 0.001	196	4.55
R AMY	21	–2	–26			4.15
*Post hoc t* tests of valence conditions						
Negative faces > neutral faces						
R medial temporal pole	45	10	–36	*p* < 0.0001	511	5.53
L anterior cingulate cortex	–3	50	–2	*p* < 0.0001	1254	5.40
R superior temporal gyrus	49	–6	–4	*p* < 0.0001	393	5.26
L temporal pole	–35	20	–22	*p* < 0.0001	1124	5.07
L AMY	–19	–6	–24			5.05
R parahippocampal gyrus	27	–20	–22	*p* = 0.0003	325	4.99
R AMY	21	0	–28			4.47
L fusiform gyrus	–21	–52	–16	*p* < 0.0001	791	4.67
R paracentral lobule	9	–32	58	*p* = 0.015	173	4.55
R paracentral lobule	11	–44	66	*p* = 0.04	138	4.26
L inferior frontal gyrus	–39	32	2	*p* = 0.037	141	4.24
L middle frontal gyrus	–27	16	52	*p* = 0.037	141	4.14
L superior temporal gyrus	–55	–10	–4	*p* = 0.028	150	3.95
L superior medial gyrus	–9	38	50	*p* = 0.036	142	3.78
Positive faces > neutral faces						
L lingual gyrus	–19	–66	–6	*p* = 0.043	136	4.59
Negative faces > positive faces						
L inferior temporal gyrus	–45	2	–34	*p* = 0.048	132	4.42

Significant clusters on whole-brain level in the second-level contrast face matching versus shape matching, the ANOVA across valence conditions, and *post hoc t* tests. Significance level at whole-brain cluster-level threshold *p*_FWEc_ < 0.05, cluster-defining threshold at *p*_CDT_ < 0.001. k, cluster size; R, right; L, left.

### DCM

#### Family-wise model comparison

**Figure 4.**
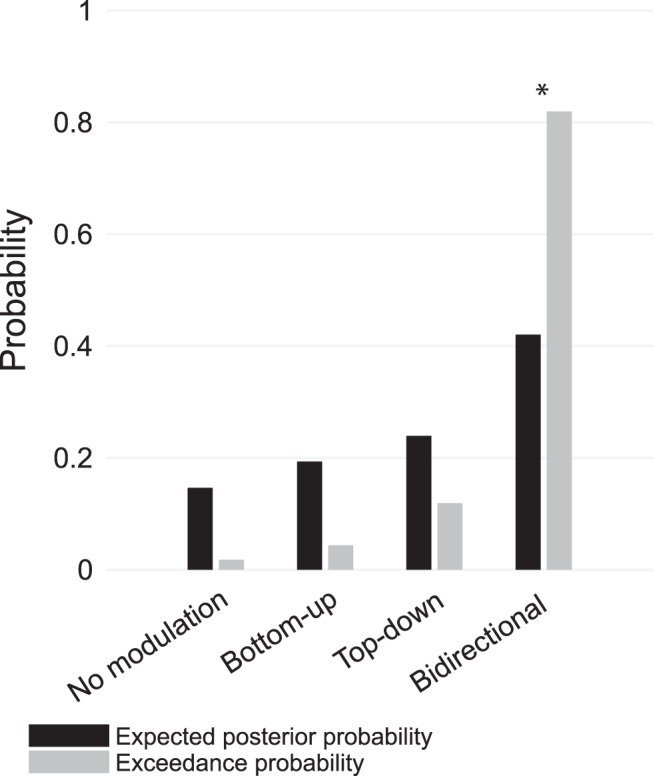
Family-wise Bayesian model comparison. Expected posterior probabilities and exceedance probabilities for the four specified model families. Asterisks (*) indicate the winning model family.

In a first step, we compared different model families (Fig. 4). The model family with bidirectional modulations of connections outperformed all other families with an expected posterior probability of 42% and an exceedance probability of 82%. As the model space incorporated a wide range of plausible models, we subsequently performed Bayesian model averaging to infer on the model parameters of the winning family.

#### Bayesian model averaging

**Figure 5.**
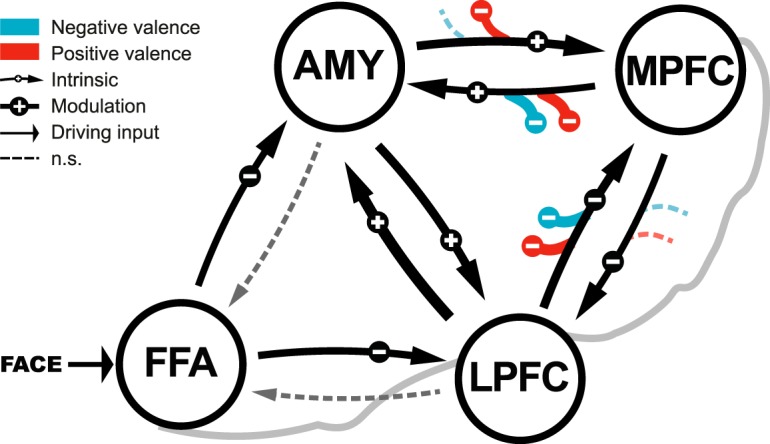
Effective connectivity during face-matching and its modulation by positive and negative valence. Parameters have been averaged with Bayesian model averaging, across all subjects and models of the winning model family. We found significant dampening of effective connectivity from the AMY to the MPFC during processing of positively valenced faces. Arrow thickness indicate effective connectivity values: thick > 0.15, medium > 0.10, thin > 0.05, dashed: not significant.

The results from Bayesian model averaging (BMA; Table 3; Fig. 5) emphasize the relevance of connections between the AMY and the MPFC during processing of emotional faces. One-sample *t* tests for consistency across subjects showed that the average endogenous connectivity is significant between those regions. In addition, we found significant modulation of connectivity by valence on these connections. While the effective connectivity from the MPFC to the AMY was modulated by positive and negative valence, the modulation of effective connectivity of the bottom-up connection from the AMY to the MPFC was significant for the positive but not the negative condition.

**Table 3.** Mean and SD of endogenous and modulatory parameter estimates for all connections across all subjects and across the models of the winning bidirectional family, and the respective *p* value resulting from a one-sample *t* test (corrected for multiple comparisons)

Connection type	Mean	SD	p_FDRc_
Endogenous parameters			
MPFC → AMY	0.0727	0.0193	0.0241^*^
MPFC → LPFC	–0.0823	0.0181	0.0109^*^
LPFC → MPFC	–0.1471	0.0174	<0.0001^*^
AMY → MPFC	0.1122	0.0178	0.001^*^
AMY → LPFC	0.1207	0.0168	0.001^*^
LPFC → AMY	0.1702	0.0177	<0.0001^*^
AMY → FFA	0.0579	0.0182	0.0505
LPFC → FFA	0.0018	0.0181	0.4786
FFA → AMY	–0.0561	0.0135	0.0325^*^
FFA → LPFC	–0.1076	0.0126	0.0002^*^
Modulatory parameters			
MPFC → AMY, positive	–0.1799	0.0698	0.01^*^
MPFC → LPFC, positive	0.0826	0.0607	0.0975
LPFC → MPFC, positive	–0.1575	0.0507	0.0021^*^
AMY → MPFC, positive	–0.1434	0.0576	0.0109^*^
MPFC → AMY, negative	–0.2732	0.0664	0.0001^*^
MPFC → LPFC, negative	0.0149	0.0625	0.4295
LPFC → MPFC, negative	–0.2174	0.0665	0.0014^*^
AMY → MPFC, negative	–0.1051	0.0724	0.0881

* Significant (FDR adjusted *p*_FDRc_ < 0.05, df = 31).

Average intrinsic connectivity between the MPFC and LPFC differed significantly from zero across subjects. In addition, the connection between the LPFC and the MPFC showed a significant modulation effect of positive and negative valence, suggesting a specific role of this connection during processing of emotional stimuli.

After averaging the intrinsic and modulatory connectivity parameters, we correlated each subject’s individual connectivity parameters with the behavioral data from the task (mean accuracy and the mean RTs for the different valence conditions). However, none of the correlations remained significant after correction for multiple comparisons.

## Discussion

Our study examined the valence-dependent functional architecture of the prefrontal-AMY network during emotion processing using statistical parametric mapping and DCM. We used a dynamic face-matching and shape-matching paradigm in healthy subjects to assess activity and connectivity in regions supporting emotion processing and, subsequently, whether emotional valence modulates effective connectivity of bottom-up (salience signals), top-down (evaluation and regulation signals), or bidirectional connections. The results of our study suggest three main conclusions.

First, we corroborated earlier studies by showing that the MPFC as a core region of emotional response regulation is especially sensitive to negative affect (Ochsner et al., 2012). Our data suggest that during processing of negative valence the MPFC and the right AMY are more strongly activated than during processing of neutral valence. Second, we directly demonstrated that activity in key regions of the prefrontal-AMY network during emotion processing is best explained by bidirectional contextual modulation of effective connectivity by valence. Accordingly, processing emotional valence directly induces changes of coupling strengths within the prefrontal-AMY circuitry. In particular, model averaging showed that the bidirectional coupling between MPFC and AMY and unidirectional coupling between LPFC and MPFC were modulated by affective cues. This suggests that the MPFC not only serves the integration of bottom-up and top-down signals, but also continuously exerts influence on the AMY during face-matching. Third, we found evidence for a differential effect of valence on coupling between regions. On the one hand, effective connectivity from the MPFC to the AMY was modulated by both positive and negative valence, while on the other hand effective connectivity from the AMY to the MPFC was only significantly modulated by positive valence. Additionally, the connectivity from the LPFC to the MPFC was augmented during positive and negative valence processing. Previous studies have highlighted the role of the MPFC during emotion processing and they have underlined the role of the MPFC in processing of valence (Kawasaki et al., 2001; Winecoff et al., 2013). Studies using explicit emotion regulation paradigms have repeatedly shown that the activation of the MPFC is increased during the reappraisal of negative emotion (Urry et al., 2006; Delgado et al., 2008). Thus, it has been suggested that the MPFC supports the control of emotional responses. Moreover, recent work proposed that the involvement of the MPFC during emotion processing is related to the encoding of an integrated affective value of a stimulus (Smith et al., 2010; Winecoff et al., 2013). Importantly, this integrated affective value encoded in the MPFC might be crucially dependent on the confidence in the aggregated information (Lebreton et al., 2015), which might modulate the BOLD signal in the MPFC following a U-shape pattern (Barron et al., 2015). Our data show significantly increased activity in the MPFC during the processing of emotional stimuli with negative valence. Our findings may therefore reflect the encoding of biological significance of negatively valenced faces and provide further evidence for the encoding of stimulus valence in the MPFC. Accordingly, the lower responses in the MPFC in the neutral condition could reflect either lower relevance or lower confidence in the nature of stimuli. This would emphasize the role of the MPFC in the integration of affective information within a valence-sensitive network, computing a value for biological significance for a given stimulus.

This is supported by our modeling results showing that the MPFC integrates affective information from multiple routes. Model averaging demonstrated that the bidirectional coupling between the AMY and MPFC and the coupling from the LPFC to the MPFC are valence sensitive. This is not only in line with recent theories of distributed processing of emotional stimuli along multiple parallel pathways (Pessoa and Adolphs, 2010), but also provides direct evidence for the idea that the prefrontal-AMY circuitry can change its functional state to support appropriate mental functions for a given context (Pessoa, 2017), which potentially requires action.

Bayesian model averaging showed that positive valence significantly dampened the intrinsic connectivity between the AMY and the MPFC, whereas negatively valenced faces did not. As task difficulty between negative and positive conditions was comparable, the observed difference in connectivity strongly suggests valence-sensitivity of the coupling between these regions. That said, the similar effective connectivity of the bottom-up connectivity during neutral and negative blocks was particularly interesting to us, since the pathway from the AMY to the MPFC is thought to be specifically sensitive to negative valence (e.g., fear conditioning; for review, see Kim et al., 2011). Notably, this is the first study that investigated valence-dependent effective connectivity within the prefrontal-AMY network using dynamic faces. The similar connectivity pattern of the neutral and negative condition might stem from the AMY’s role as a significance detector. The AMY has been extensively studied and there is a broad consensus on the relevance of this brain structure in face processing (Adolphs, 2002), and more generally, the immediate detection of biological significance (Sander et al., 2003), or resolving uncertainty (Whalen, 1998). Neutrally (i.e., ambiguously) and negatively valenced faces might induce increased predictive uncertainty compared to positively valenced faces (Whalen et al., 2013). A plausible brain response to react to predictive uncertainty would be to relocate cognitive resources to resolve it (Bubic et al., 2010). The coupling between AMY and MPFC during processing neutral and negative facial expressions might therefore reflect a signal that translates into a need for action to increase precision and, hence, regain confidence in a volatile environment. In line with this, we found significant effective connectivity from the AMY to MPFC during processing of neutral and negative valence, reflected in a significant intrinsic connectivity and its non-significant modulation during negative blocks, which might reflect a bottom-up confidence signal from the AMY.

Furthermore, we found positive intrinsic connectivity from the MPFC to the AMY during the dynamic face processing that was modulated negatively during positive and negative blocks. This is in line with previous work that observed negative effective connectivity between these regions in healthy subjects using a similar task with static emotional faces (Sladky et al., 2015a) and might reflect a downregulation mechanism of automatic dampening the emotional response of the AMY by the MPFC to negative emotional cues (Ochsner et al., 2012).

Corticocortical effective connectivity between LPFC and MPFC was significant during face processing. A general valence-independent face-sensitive coupling between LPFC and MPFC in our dynamic task could reflect a cognitive attenuation of significance of emotional stimuli, which would eventually yield an adaption of emotional responses mediated by the MPFC. In this regard, the LPFC has been implicated in emotion regulation strategies such as repression (Anderson et al., 2004) or (spontaneous) reappraisal (Drabant et al., 2009). Based on our modeling results, we therefore propose that the valence-sensitive recruitment of the MPFC originates from the integration of affective information stemming from valence-dependent coupling within the prefrontal-AMY network. Our findings for the afferent connections of the LPFC are in agreement with the results reported in a previous study (Sladky et al., 2015a), that found an up-modulating effect of the AMY on the LPFC and a down-modulating effect of medial prefrontal regions over lateral ones. On the one hand, enhanced activation of the LPFC via the AMY could reflect the allocation of attentional resources toward emotionally salient stimuli of high biological significance, on the other hand, the downmodulating signal from the MPFC might support the continuous release of these resources (Bishop et al., 2004; Sladky et al., 2015a). Interestingly, we did not observe any evidence of valence sensitivity of the connection from the MPFC to the LPFC, suggesting a general downmodulating role of this connection during face-matching.

Forward connections from the FFA showed significant intrinsic connectivity to the AMY and the LPFC, while the backward connections were not. This feed-forward functional architecture during face processing has been described previously (Fairhall and Ishai, 2007), and our results confirm these findings.

One limitation regarding the interpretation of our findings are the emotional categories of the faces used in our paradigm. Unlike many previous studies (Fusar-Poli et al., 2009; Zinchenko et al., 2018), we did not use angry or fearful faces for the negative condition, but sad and disgusted faces. This choice was made deliberately to reduce the effects of arousal (Remmington et al., 2000; Trautmann et al., 2009). Thus, our findings should be only interpreted in regard to the emotional expressions used in our paradigm. Despite this limitation, our results are in concordance with previous findings in the literature and provide further evidence that the state of the prefrontal-AMY network is sensitive to valence. The goal of future investigations should be to assess whether our results are generalizable to other negative emotional expressions and to negatively valenced stimuli, other than faces, in general.

To conclude, using DCM analysis we showed valence-dependent coupling changes within the emotion processing circuitry during a dynamic face-matching paradigm. Our findings are in agreement with recent theories of affect processing that stress the highly dynamic nature of network interactions. It has been suggested that these interactions do not only depend on task-demands, but, as our empirical data suggest, on the emotional valence of a stimulus (Kim et al., 2004; Pessoa, 2017). Understanding mechanisms of dynamic integration of affective value in the emotion processing network might be pivotal for explaining psychopathologies. A dysregulation of the prefrontal-AMY network has been found in various psychiatric disorders. A disruption of neural circuitry underlying successful emotion regulation is a hallmark of various psychiatric conditions such as mood and anxiety disorders in adults (Johnstone et al., 2007; Almeida et al., 2009b; Etkin et al., 2010; Liao et al., 2010; Demenescu et al., 2013; Sladky et al., 2015a; Minkova et al., 2017) and adolescents (Monk et al., 2008; Perlman et al., 2012; Keshavan et al., 2014) and dysfunctional valence-dependent coupling might underlie the attention and processing bias in mood disorders (Disner et al., 2011; Groenewold et al., 2013; Clark et al., 2018). Our study provides strong evidence for alterations of coupling as a function of valence within the prefrontal-AMY network. Based on our results, such a dynamic face-matching task thus may aid future studies to probe and disentangle mechanisms of attentional bias and valence-sensitive emotional dysregulation in neuropsychiatric disorders.

## Synthesis

Reviewing Editor: Daniela Schiller, Mount Sinai School of Medicine Decisions are customarily a result of the Reviewing Editor and the peer reviewers coming together and discussing their recommendations until a consensus is reached. When revisions are invited, a fact-based synthesis statement explaining their decision and outlining what is needed to prepare a revision will be listed below. The following reviewer(s) agreed to reveal their identity: Dylan Gee, Xiaosi Gu.

This study used dynamic causal modeling of fMRI data collected during a face emotion task in healthy controls to examine prefrontal-amygdala circuitry and its modulation by emotional valence in healthy adults. The authors report that bidirectional prefrontal-amygdala coupling was increased by emotional valence, and that negative (but not positive) valence enhanced bottom-up connectivity from the amygdala to the mPFC. Strengths include the use of dynamic stimuli and the study conceptualization, which is well-grounded in the current literature. These findings are interesting and provide amongst the first insights into the directional relationship between amygdala and prefrontal regions under the modulation of emotion. Ultimately, these findings would be important for interpreting the emotional deficits found in many psychiatric disorders. If the methodological limitations raised can be addressed, the results of this study provide important information about the nature of prefrontal-amygdala interactions (e.g., the role of mPFC, modulation by valence, etc.) that have not been addressed in previous research.

Specific comments:

1) Motion is a known confound in studies of connectivity (e.g., Power et al., 2012), including task-based connectivity (e.g., Siegel et al., 2014). There does not seem to be any mention of criteria employed for exclusion based on motion in the current manuscript. How did the authors control for motion? Were any volumes or participants excluded due to excessive motion? Even if motion was very low, it should be characterized (e.g., mean motion in each direction, mean framewise displacement, etc.) so that the reader has a clearer picture of motion in this sample. Without this information, it is not possible to evaluate the current methods or results.

2) Given that the primary focus of the study is on valence, the operationalization of valence is particularly important. It is unclear how the authors selected which emotional faces to include in which blocks, and the results must be interpreted more specifically in the context of the faces that were included. There are two primary concerns- one is that surprised faces were included in both the positive and neutral blocks, and not in the negative blocks, while existing research shows strong evidence that the valence of surprised faces is largely dependent on individual differences (e.g., Neta et al., 2009; Neta et al., 2011; Neta et al., 2017). The other is that the negative valence block consists only of sad and disgusted faces, whereas the majority of extant literature (particularly in anxiety, which the authors reference) is based on angry and fearful faces.

3) The sample size (N=33) is relatively small and seems quite arbitrary. What was the target sample size? What was the authors' stopping rule?

4) How were psychiatric disorders evaluated for exclusion?

5) Were individual connectivity parameters associated with activation? One might expect stronger top-down connectivity to be associated with lower amygdala activation, and this dataset has the potential to inform this open question.

6) The whole brain GLM is not clearly described. How many actual events were included as regressors? In order to model driving and modulatory inputs, each input should be included as a separate event regressor (e.g. one for ‘all stimuli’, one for ‘all faces’, one for ‘all negative faces’ etc.)

7) The behavioral results are interesting and deserve being in the figure (e.g. as fig 1b).

8) The discussion on pg. 18 of parallels between the current findings and those in patients with anxiety is highly speculative - this should be emphasized given the absence of any associations with anxiety in the current sample. Alternatively, do the authors have a measure of trait anxiety that they could examine in this sample?

9) Line 191: “... the behavior of specific brain regions” sounds confusing - do the authors mean the activities of specific brain regions?

10) DCM analysis:

- The study could examine intrinsic connection between amygdala and LPFC - It does not require direct anatomical connections.

- Driving input - do the authors mean the driving input is all events (face and shape combined)? It is possible that using a visual ROI as the entrance of all visual stimuli would be more appropriate and/or change the results. Have the authors considered such possibility?

- To consider neutral as a valence seems counterintuitive. A more direct and comprehensive way to examine the impact of valence might be to construct DCMs with positive (against neutral) and negative (against neutral) as two modulatory inputs, while using both all visual stimuli and all faces as two driving inputs entering at the level of visual ROIs (e.g. V1 and FFA, respectively; resulting a total of 5 ROIs in the models). A slightly simpler version of this 5-region DCM would be to focus on face stimuli (while still including shape trials in the GLM): driving input - all faces, entering FFA; modulatory input 1 - negative faces; modulatory input 2 - positive faces, etc.

- The DCM result of equivalent impact of negative and neutral faces on AMY-MPFC connectivity is difficult to interpret and does not fit into the literature of the distinct neural effects of negatively valenced stimuli in general. It is unclear whether these results would still hold if the authors revise the DCM to include proper visual ROIs for driving inputs.

- Did the authors measure any traits related to mood or other disorders (e.g. BDI, anxiety etc.) and if so, are there any correlations between these measures with the DCM estimates?
